# Ultra-Sensitive Detection of Bacterial Spores via SERS

**DOI:** 10.1021/acssensors.4c03151

**Published:** 2025-01-23

**Authors:** Jonas Segervald, Dmitry Malyshev, Rasmus Öberg, Erik Zäll, Xueen Jia, Thomas Wågberg, Magnus Andersson

**Affiliations:** † Department of Physics, Umeå University, Umeå SE-901 87, Sweden; ‡ Wallenberg Initiative Materials Science for Sustainability, Department of Physics, Umeå University, Umeå SE-901 87, Sweden

**Keywords:** plasmonics, spores, DPA, nanorods, SERS, detection

## Abstract

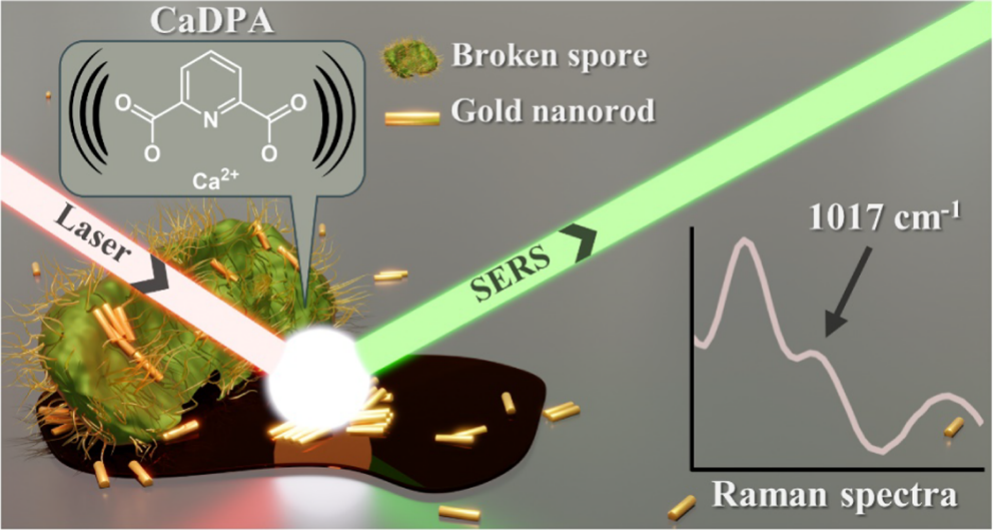

Bacterial spores are highly resilient and capable of surviving extreme conditions, making them a persistent threat in contexts such as disease transmission, food safety, and bioterrorism. Their ability to withstand conventional sterilization methods necessitates rapid and accurate detection techniques to effectively mitigate the risks they present. In this study, we introduce a surface-enhanced Raman spectroscopy (SERS) approach for detecting *Bacillus thuringiensis* spores by targeting calcium dipicolinate acid (CaDPA), a biomarker uniquely associated with bacterial spores. Our method uses probe sonication to disrupt spores, releasing their CaDPA, which is then detected by SERS on drop-dried supernatant mixed with gold nanorods. This simple approach enables the selective detection of CaDPA, distinguishing it from other spore components and background noise. We demonstrate detection of biogenic CaDPA from concentrations as low as 10^3^ spores/mL, with sensitivity reaching beyond CaDPA levels of a single spore. Finally, we show the method’s robustness by detecting CaDPA from a realistic sample of fresh milk mixed with spores. These findings highlight the potential of SERS as a sensitive and specific technique for bacterial spore detection, with implications for fields requiring rapid and reliable spore identification.

Bacterial spores are notorious for their extraordinary resilience, allowing them to endure harsh environmental conditions and persist in diverse habitats, such as soil and solid surfaces for extended periods, ranging from years to decades. They also survive traditional cleaning and disinfection methods which typically kill vegetative cells, presenting significant challenges for various sectors, including food and beverage, healthcare, and public safety. For instance, contamination by *Bacillus cereus* (*B. cereus*) spores leads to food and beverage spoilage, resulting in substantial economic losses due to wasted products and the need for extensive cleaning procedures.^1^ In healthcare settings, *Clostridioides difficile* (*C. difficile*) spores cause dangerous hospital-acquired infections, necessitating costly and time-consuming disinfection and decontamination measures to control.^2,3^ Additionally, *Bacillus anthracis* (*B. anthracis*) spores remain a major public health threat, especially in rural areas of developing countries where outbreaks are harder to control. Their resilience and potential use in biological warfare or terrorism further amplify the threat.^4^ Given spores’ widespread impact, the development of rapid and accurate field detection methods is essential to limit their spread, reduce associated costs, and address related health concerns effectively. Several detection methods are available to evaluate spore contamination levels, each with their strengths and limitations. A traditional approach involves culturing samples on selective agar to quantify spore content, a method known for its reliability and sensitivity.^5,6^ While effective, this technique is time-consuming, often requiring 12 h of incubation or longer, and demands access to specialized laboratory facilities. Immunoassays are an alternative method, which utilize enzyme-linked immunosorbent assays and offer high accuracy in spore detection.^7^ The method achieves its high accuracy by employing specific antibodies that bind to targeted antigens, but requires highly trained personnel and expensive reagents.^8^ Polymerase chain reaction (PCR) assays, often considered the “gold standard” for pathogen detection, provide high sensitivity and specificity by amplifying target DNA or RNA sequences. However, PCR-based environmental sampling of spores is complicated by the difficulty of lysing spores to release their DNA, particularly when compared with vegetative cells.^9,10^ More recent methods involve using nanoparticles functionalized with antibodies or short complementary DNA strands for spore detection, enabling simple techniques like colorimetry and lateral flow assays to detect spores directly with high specificity.^11,12^ Despite their promise, these methods may require long development times as the nanoparticles must be custom-designed and optimized for each species tested.

Spectroscopic techniques are an alternative approach, offering rapid, nondestructive detection of biological molecules with exceptional specificity and sensitivity. Among these techniques, Raman spectroscopy stands out for its versatility and label-free nature, making it attractive for researchers and practitioners alike.^13^ It identifies molecules by measuring the vibrations of their chemical bonds during laser exposure. These vibrations cause Raman scattering, which produces a unique spectrum to each molecule, acting as a molecular fingerprint. This unique spectrum allows for detection, quantification, and differentiation between biochemically distinct substances. However, conventional Raman spectroscopy often suffers from weak signals due to the inherent low cross section for Raman scattering. Surface-enhanced Raman spectroscopy (SERS) overcomes this limitation by significantly amplifying the Raman signal by utilizing the strong electromagnetic field near metal nanostructures.^14^ These fields are induced by the resonant oscillation of free electrons, known as localized surface plasmon resonance (LSPR), in response to incoming light. The degree of amplification is proportional to the fourth power of the field’s amplitude, meaning even small changes in the local field result in substantial signal enhancements.^15^ Additionally, chemical enhancement occurs through charge transfer between the adsorbed molecules and the metal surface, further amplifying the Raman signal. Together, these enhancements dramatically increase the Raman signal, enabling the detection of even single molecules.^16^ SERS also provides the benefits of rapid and straightforward detection without the need for labeling, making it especially suitable for field measurements.^17^ However, as bacteria contain a large variety of chemical structures, the resulting Raman signals are often complex, and the locally acting surface enhancement (<3 nm) can yield large variations in signal within the same bacterial sample.^18^ Therefore, effective use of label-free SERS in detection assays requires a thorough understanding of specific Raman-active biomarkers that can be reliably distinguished.

In the context of detecting bacterial spores by SERS, calcium chelate dipicolinic acid (CaDPA) stands out as the most reliable and specific biomarker, generating a distinct Raman peak around 1017 cm^–1^.^19,20^ Although some studies aim to detect spores by their surface fingerprint,^21^ the unique presence of CaDPA in spores makes it a reliable biomarker to target. However, given that biogenic CaDPA is located within the spore core, positioned over 100 nm from its surface, extraction of CaDPA is essential for SERS detection.^22^ A commonly used method for extracting CaDPA involves treating spores with nitric acid combined with ultrasonication.^17,23,24^ Zhang et al.^17^ reported a 34% extraction efficiency using this approach, with a limit of detection (LOD) of 10^7^ spores/mL using a silver film over nanospheres substrate. Similar detection limits have been reported in other studies (10^6^ spores/mL),^22,23^ while more recent research literature reports detection limits of 10^4^ spores/mL, with a sensitivity of ∼100 spores.^24^ In terms of detecting pure dipicolinic acid (DPA), the unchelated form of CaDPA, SERS has demonstrated remarkable sensitivity, with reported levels corresponding to less than one spore.^25,26^ However, demonstrating this sensitivity with biogenic CaDPA is significantly more challenging. Issues like efficient CaDPA extraction from the spore core, interference from biological material and reliable peak identification complicate the detection process.

In this study, we used probe sonication to extract CaDPA from *Bacillus thuringiensis* (*B. thuringiensis*), a model organism for bacterial spores, achieving an extraction efficiency of ∼75%. We then utilize SERS with gold nanorods to enhance the Raman signal of CaDPA to detectable levels, using a simple drop-drying method. To confirm the specificity of the targeted 1017 cm^–1^ peak, we compared the results to a CaDPA-deficient mutant strain, validating that the detected signal originated from CaDPA rather than other organic materials. Our results demonstrate the successful detection of nonpurified CaDPA in complex samples containing disrupted spore constituents, with a clearly distinguished signal from other spore materials and noise sources.

Notably, we demonstrate detection down to 10^3^ spores/mL, highlighting our method’s ability to detect levels below infectious doses of many pathogenic spore-forming bacteria. For example, *B. anthracis* and *B. cereus* can be infectious at doses as low as 10^4^ colony-forming units (CFU).^23,27^ To further validate our method, we tested it using a realistic sample of milk mixed with spores, achieving detection at a concentration of 10^6^ spores/mL. Despite using a nonoptimized protocol, this detection level remain suitable for industrial applications, as typical infectious levels of *B. cereus* in food range from 10^5^ to 10^8^ CFU/g.^28^ Additionally, our method demonstrated a sensitivity to sub-spore levels of CaDPA, indicating that refinement of the methodology could further enhance the detection limit. Overall, this study highlights the robustness and potential of using SERS for rapid and sensitive detection of bacterial spores, with applications in healthcare, the food industry, and environmental monitoring.

## Experimental Section

### Nanoparticles and Materials

Gold nanorods, with specified dimensions of 20 nm in diameter and 95 nm in length, were purchased at a concentration of 9.69 × 10^10^ particles/mL (NR-20–780–50, NanoSeedz Limited, China). Their actual dimensions were verified as 72 ± 7 nm in length and 20 ± 4 nm in diameter, based on an average of 20–25 measurements from the transmission electron microscopy (TEM) image in Figure S1, using the Fiji (ImageJ) software.^100^ They were provided in a liquid dispersion with the surfactant cetyltrimethylammonium bromide (CTAB) to prevent particle agglomeration. The nanorods were concentrated by a factor of 10 through centrifugation at 5000*g* for 10 min, followed by removal of the liquid portion (supernatant) remaining above the sedimented nanorods. We deposited all SERS samples on polished silicon wafers (BW14036, Siegert Wafer GmbH).

For testing the detection method, we utilized *B. thuringiensis* ATCC 35646 spores, with original stock provided by the Swedish Defense Research Agency (FOI), Umeå. To validate the specificity of the SERS signal for CaDPA-abundant spores, we employed a*Bacillus subtilis* str. 168 DPA-deficient mutant strain (ΔspoVAC:lox72 ΔsleB:erm ΔgerA:*Cm*) as a CaDPA-negative control. The strain was provided by David Rudner, Harvard University. Spore stocks were cultured on TSA agar (Bacto, BD) at 37 °C for 1 week to induce sporulation. After growth, spores were harvested from the plates, suspended in distilled water (Synergy, Merck Millipore) and washed three times through centrifugation (5000*g*, 5 min). The resulting stock was stored in distilled water at 4 °C until use. Spore concentration was evaluated by drop counting colonies on an agar plate. Ahead of use, the spore suspension concentration was adjusted to 10^9^ spores/mL and mixed thoroughly by vortexing at 3000 rpm (VM3 Vortex, M. Zipperer GmBH) for 15 s.

### Electron Microscopy

All scanning electron microscopy (SEM) images were done on 0.1–1 μL spore suspension drops, air-dried on a glass slide. Ahead of imaging, we coated the sample with a 5 nm layer of platinum using a Quorum Q150T-ES sputter coater (Quorum Technologies). We then imaged spores, nanoparticles and supernatant using a Carl Zeiss Merlin FESEM electron microscope using the InLens imaging mode at magnifications up to 100,000×. For electron diffraction spectroscopy (EDS), we used an X-Max 80 EDS detector (Oxford Instruments), with the Aztec 3.3 software (Oxford Instruments), operating at 7 kV and 300 pA. The supernatant map was acquired with a dwell time of 10 ms, with other maps being done at 6 ms.

To prepare spores for TEM, we fixed the spore suspensions using 2.5% glutaraldehyde (TAAB Laboratories, Aldermaston, England) in 0.1 M PHEM buffer, and then postfixed them in 1% aqueous osmium tetroxide. To further dehydrate the spores, we washed them in ethanol and acetone, after which they were embedded in Spurr’s resin (TAAB Laboratories, Aldermaston, England). The 70 nm spore sections were then post contrasted in uranyl acetate and Reynolds lead citrate ahead of imaging. We imaged the spores using a Talos L120C (FEI, Eindhoven, The Netherlands) operating at 120 kV. Micrographs were acquired using a Ceta 16 M CCD camera (FEI, Eindhoven, The Netherlands), equipped with TEM Image and Analysis software v. 4.17 (FEI, Eindhoven, The Netherlands). For TEM of Au nanorods, they were directly deposited and dried on a copper Formvar grid (Ted Pella, U.S.) before imaging.

### Laser Tweezer Raman Spectroscopy

Raman spectra from individual spores were obtained using an in-house laser tweezer Raman spectroscopy (LTRS) setup with an inverted Olympus IX71 microscope, as outlined in Stangner 2018,^101^ Dahlberg 2020.^102^ The system employs a 785 nm laser (08-NLD, Cobolt), adjusted to 60 mW at the sample. Focusing is through a 60× (UPlanSApo, Olympus) water immersion objective with a 1.2 numerical aperture. Raman signals, filtered by a 785 nm notch filter (Thorlabs NF785-33), are analyzed with a McPherson 207 spectrometer with a 600 grooves/mm grating (800 nm blaze) and a Peltier-cooled CCD detector (Andor Newton 920N-BR-DDXW-RECR) at −95 °C. The sample is positioned on a temperature-controlled stage set at 25 °C to stabilize intensity and prevent fluctuations due to temperature changes during measurements. The instrument achieves a spectral resolution of less than 3 cm^–1^. Spectrum acquisition and detector control are facilitated using Solis software (v. 4.30, Andor).

### Absorbance of Au Particles

In addition to the previously mentioned nanorods, some additional gold particles were considered for the SERS measurements, all from the same manufacturer (NanoSeedz Limited, China). This includes nanospheres with 100 nm diameter (NS-100-50), nanocubes with 50 nm sides (Nanocube-90) and nanobipyramids measuring 35 nm in diameter and 110 nm in length (NBP-35-815-20). All nanoparticles were delivered in dispersions with the surfactant CTAB. All particle absorbance measurements were done in dispersions using a UV/vis (PerkinElmer Lambda 1050) spectrophotometer, covering a range of 250 to 1200 nm. These measurements were performed in quartz cuvettes, with a step length and slit width of 5 nm, incorporating both 100% and 0% transmittance for baseline correction. Prior to the absorbance measurements, the particle dispersions were diluted 5× with deionized water. A 1 mM solution of CTAB with 99% purity (11446801, Fischer Scientific) was further prepared to assess its impact on the absorbance spectra of the particle dispersions.

### Spore Sonication and Sample Preparation

One mL of spore suspension in a 1.5 mL Eppendorf tube was subjected to probe sonication using a 20 kHz, 130 W ultrasonic processor (VCX 130, Sonics & Materials, Inc.), equipped with a 3 mm probe (630–0422, Sonics & Materials, Inc.). The probe was inserted to a depth of 1 cm into the liquid, without touching any walls. The program cycle was set to 5 s on and 5 s off at 80% amplitude for 30 min. To prevent sample overheating an aluminum holder was used as heat sink and 2 intermittent pauses of 1 min were included (total effective sonication time, 15 min). After sonication, we centrifuged the sonicated spore sample at 16000*g* for 10 min to pellet the remaining nonsoluble debris. We collected the supernatant and stored it at 4 °C, diluting it in distilled water as necessary for experiments.

Samples were prepared by mixing a 1:1 ratio between the concentrated nanorod stock and supernatant solution, diluted as necessary. We then dropped 1 μL of this mixture on a polished silicon wafer and allowed it to air-dry at room temperature. For experiments involving the supernatant covering the nanoparticles, 1 μL of nanoparticles was first drop dried on the wafer, followed by an additional 1 μL of spore suspension, allowing it to dry on top of the nanoparticle layer.

Photographs of the dried droplets were taken using a Sony ILCE6600 camera with a 6000 × 4000 pixel sensor, mounted on a Leica MZ6 stereo microscope with 4× magnification.

### SERS Detection of CaDPA

All SERS measurements were done using an Invia Raman microscope (Renishaw plc), utilizing the integrated WiRE 3.4 software. Samples were excited by a continuous 785 nm laser (HPNIR785, Renishaw plc), set at 25 mW for an exposure time of 3 s, using either an air immersion 100×/0.85 NA or N PLAN EPI 50×/0.75 objective (566073, 11566072, Leica Microsystems) with a 1200 l/mm grating. For each measurement, data was collected over 10 accumulations. The Raman mapping was performed over a 19 × 32 grid using the same settings. For LOD experiments, we drop dried either 0.1 or 1 μL of spore supernatant mixed with gold nanorods, sonicated at concentrations of 10^4^, 10^3^ and 10^2^ spores/mL in a similar manner as above, using 20 accumulations.

### HPLC Detection of CaDPA

To independently confirm that CaDPA was released from the spores during sonication of the spore stock (10^9^ spores/mL), we performed high-performance liquid chromatography (HPLC) on a diluted supernatant sample and compared it to purified DPA solutions of 10 μM and 100 μM as controls (Figure S2). The CaDPA concentration was measured using an Agilent HPLC system with an autosampler, using a C18 column (250 mm × 4.6 mm i.d., 5 μm), and a UV detector operating at 270 nm, following the method described by Luo et al.^29^ The mobile phase consisted of water/acetonitrile (80:20 v/v) with 0.1% formic acid, flowing at 0.9 mL/min in isocratic mode.

### Detection of Spores in Milk

For the validation of the detection method in a realistic sample, 10 μL of 10^8^ spore suspension were added to 990 μL of commercial, refrigerated, pasteurized and nonskimmed (3% fat) milk (Norrmejerier, Sweden) for a final spore concentration of 10^6^ spores/mL. This suspension was directly sonicated as described above and then diluted 1000 times to decrease interference from milk components. Sample preparation was as described above, using 0.1 μL drops of diluted milk on a polished silicon wafer.

### Data Processing and Analysis

For the processing of our Raman spectra, we utilized an open-source script for MATLAB (v. 2020b, MathWorks), which is made available by the Vibrational Spectroscopy Core Facility at Umeå University.^30^ This script facilitates the subtraction of background noise and the correction of the baseline in Raman spectra through an asymmetrical least-squares algorithm.^31^ The parameters for this algorithm are specifically chosen to optimize the processing, with λ = 10^5^ and *p* = 10^–3^ used for our data.

All Raman data plots and additional data processing, such as averaging multiple Raman curves, were performed using Origin 2021 (OriginLab). Where smoothed graphs were used, we employed the Savitzky-Golay method, with points of window at 4 and polynomial order of 1.

To quantitatively assess the results using an industrial LOD in line with EP17 guidelines,^32^ we utilize a minimum of 60 measurements spread equally over 5 droplets, following the SERS protocol presented above but with 20 accumulations. We then evaluate the area in the region of 1014–1020 cm^–1^ with these 2 points as baseline, using the Integrate tool in Origin. The kernel density calculations were performed using the *kde* function in MATLAB (R2024a) with a bandwidth of 250. Following the EP17 guidelines, we estimate the limit of blank (LOB) using the mean and respective standard deviation of a blank sample as LOB = mean_blank_ + 1.645 SD_blank_.

### Density Functional Theory

To calculate the binding energy between the DPA molecule and a gold structure, we used density functional theory (DFT) at the B3LYP^33−35^/ LANL2DZ^36^ level of theory as implemented in Gaussian 16 v. C.01,^37^ with an ultrafine integration grid. The DPA molecule and gold geometries were optimized independently before reoptimizing the combined geometries in 8 separate configurations. Binding energies were calculated as the difference between the optimized energy of the combined geometry and the sum of the component energies.

## Results and Discussion

Our spore detection strategy centers on targeting CaDPA, the most abundant chemical in spores, constituting up to 15% of their dry weight.^38,39^ CaDPA is uniquely found in spores and plays a key role in protecting the spore core from heat, particularly under wet conditions. Given its abundance and exclusive presence within spores, CaDPA serves as a specific biomarker for spore detection.^40^ However, as CaDPA is found in all bacterial spores, it cannot be used to distinguish between spore species, meaning it cannot differentiate between pathogenic or nonpathogenic spores. In contrast, DNA-based methods, such as PCR and DNA probes, do provide species-specific identification, though they tend to be more complex and time-consuming. In routine screening applications, such as in the food industry, detecting the presence of any spores is usually sufficient, as even nonpathogenic spores can cause spoilage or disease.^41^ Therefore, CaDPA detection is a suitable method when confirming the presence of spores is more important than directly identifying their specific species, as in the milk industry.

**Figure 1:**
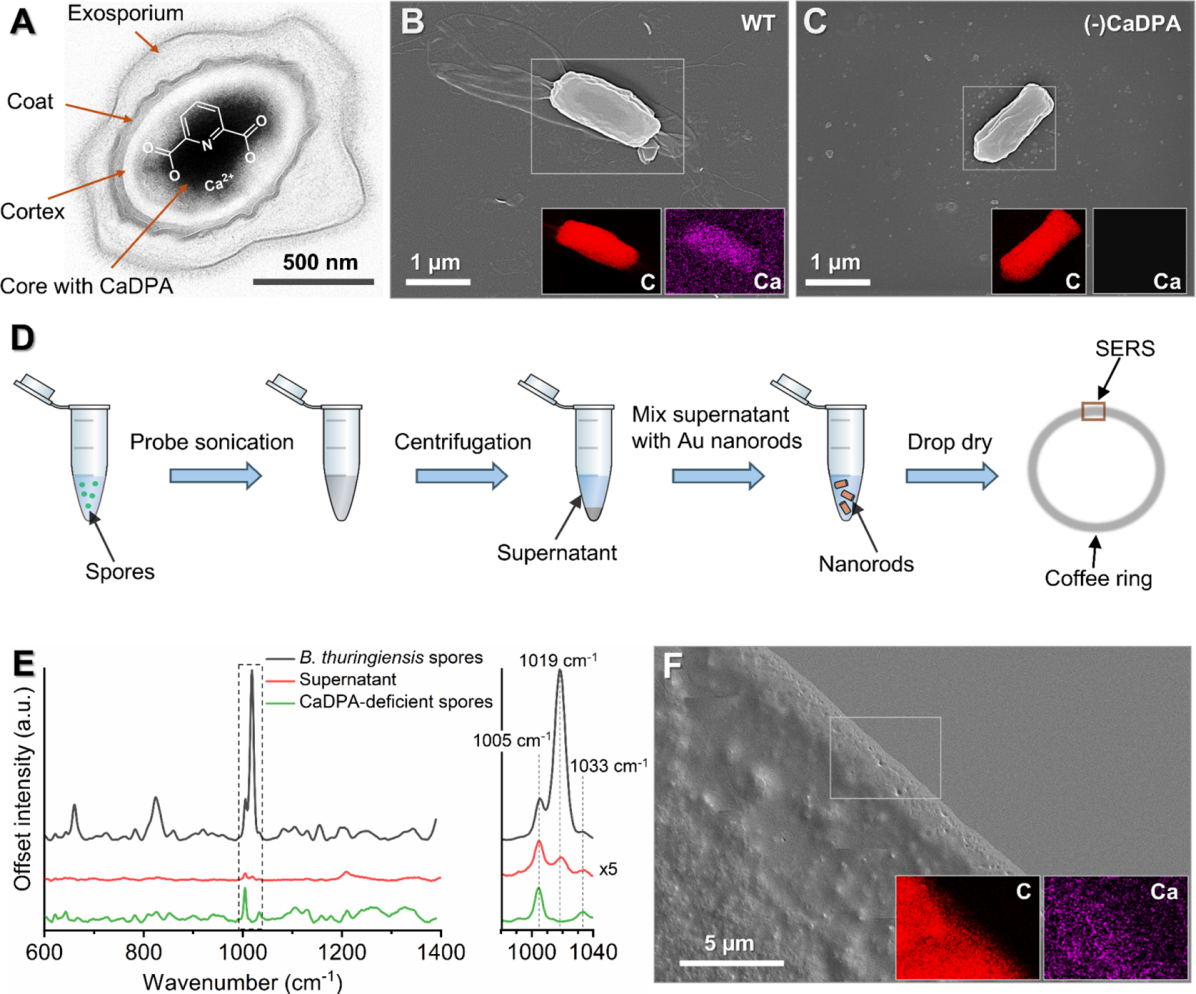
(A) TEM-based schematic showing an intact spore, with its key layers labeled, including the CaDPA-rich core. An illustration of the chemical structure of CaDPA is visible across the core. In (B) and (C) SEM images showing a top-down view of, in order, a wildtype (WT) *B. thuringiensis* spore and a CaDPA-deficient spore mutant. The EDS insets in each image show the elemental presence of carbon and calcium from each marked region. (D) Schematic of the method to release CaDPA from spores with subsequent sample preparation steps before SERS detection. (E) LTRS spectra of *B. thuringiensis* spores (black line), the supernatant from disrupted *B. thuringiensis* spores (red line, signal multiplied ×5 in the inset), and CaDPA-deficient mutant spores (green line). Each spectrum represents an average of 30 individual spectra. F) SEM image of dried supernatant with similar EDS insets of carbon and calcium.

In Figure 1A, a TEM image illustrates a cross-section of a spore, emphasizing its CaDPA-rich core amidst other structural components, such as the surrounding exosporium, coat and inner core.^42^ A SEM image, showing a top-down view of a *B. thuringiensis* spore, is presented in Figure 1B, with two insets displaying EDS maps. More specifically, the maps highlight the presence of carbon and calcium atoms, where the existence of the latter serves as an indication of the presence of CaDPA within the spore. This is further supported in Figure 1C, which shows a CaDPA-deficient mutant spore (*B. subtilis* ΔspoVAC ΔsleB ΔgerA), exhibiting similar structural integrity of a CaDPA-containing spore but lacking detectable levels of calcium.^42^ To release and detect CaDPA from the spore core, we employed the method outlined in the schematic diagram presented in Figure 1D. Initially, we subjected a concentrated *B. thuringiensis* spore stock (10^9^ spores/mL) to probe sonication, resulting in approximately 75% of the spores releasing their CaDPA content. The number of spores which release CaDPA was estimated using LTRS, where 30 optically trapped spores were examined for the presence or absence of the characteristic CaDPA Raman peak (Figure S3), typically found in the range of 1013–1020 cm^–1^.^43−45^ As CaDPA typically requires a calcium-rich medium to prevent dissociation into DPA and calcium ions (Ca^2+^) in aqueous solution, we used the similar nonchelate DPA as a reference and further validated the release of CaDPA using HPLC. We compared sonicated supernatant to pure DPA controls (10 μM and 100 μM) and estimate the concentration of CaDPA in the sonicated spore suspension to be 2.5 mM. Assuming CaDPA, with a molecular weight of 205 g/mol, comprises a conservative 10% of the spores’ mass (reported as 1.8 × 10^–11^ g per spore),^24^ this concentration corresponds to ∼3 × 10^8^ spores. This is in line with the estimate 10^9^ starting spores by drop counting.

As illustrated in Figure 1E, spores containing CaDPA exhibit a prominent Raman peak at 1019 cm^–1^, corresponding to the ring breathing mode of CaDPA. In comparison, the CaDPA-deficient mutant spore lacks this peak in its Raman spectra, indicating its reliability as a marker for the presence of CaDPA. Additionally, a weak but identifiable signal at this peak is observed in the supernatant of the centrifugated spore suspension, indicating that the liquid portion above the sedimented spores still contains some molecules or compounds of interest. This observation, combined with EDS mapping of the dried supernatant, which revealed a weak but discernible calcium signal across the dried film (Figure 1F), collectively indicates the release of CaDPA into the solution postsonication. Furthermore, limitations of traditional Raman spectroscopy become apparent, as even with a sensitive detection technique like LTRS, the 1019 cm^–1^ peak from the supernatant only appears faintly. To overcome this limitation and enhance the signal, we mixed the liquid supernatant with SERS-active gold nanorods. We then conducted SERS measurements around a drop dried “coffee ring”, resulting in significant improvement in detection sensitivity.

### Enhancing the Raman Signal by Gold Nanorods

**Figure 2:**
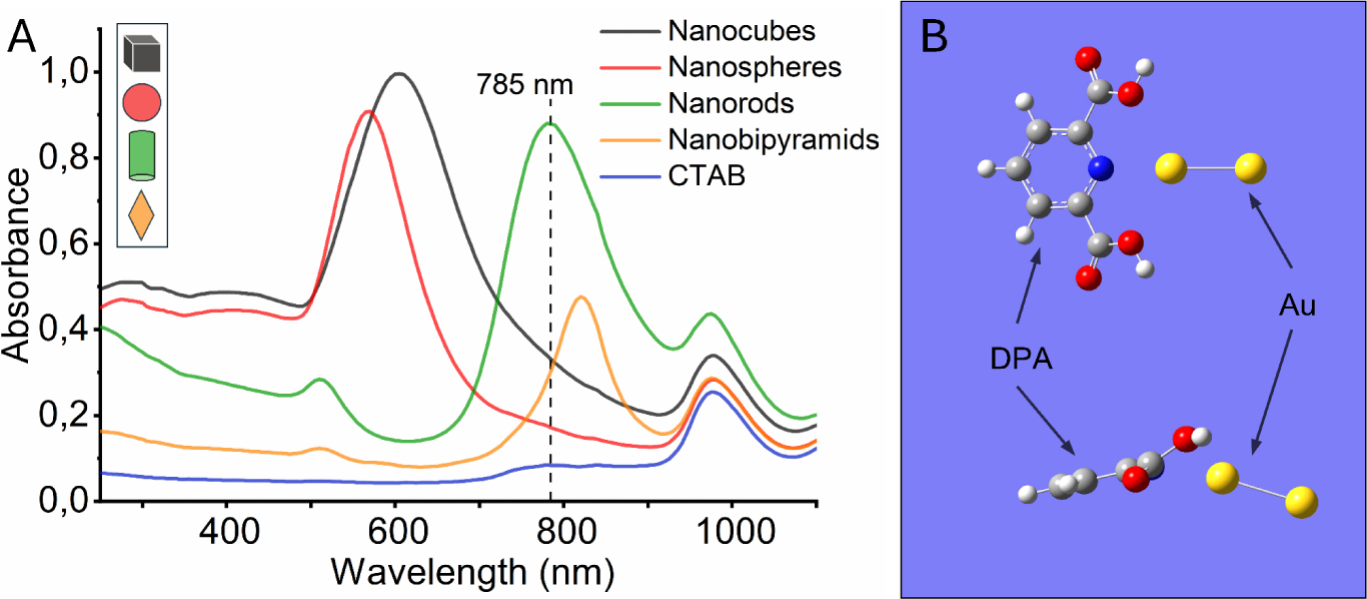
(A) Absorbance spectra of nanorods, nanospheres, nanocubes and nanobipyramids dispersed in deionized water with CTAB as surfactant. The absorbance maxima of nanorods are located at 785 nm, which matches the wavelength of the diode laser used for the SERS measurements. CTAB has no significant absorbance at 785 nm. (B) Top-down and side views of the optimized geometry from the DFT calculation, showing the configuration with the highest binding energy between the DPA molecule and the gold structure. In the visualization, the white, gray blue, red, and yellow spheres represent hydrogen, carbon, nitrogen, oxygen, and gold, respectively. The gold structure is positioned near the nitrogen atom of the central DPA pyridine group, situated between the carboxyl groups.

SERS aims to amplify Raman signals by orders of magnitude by leveraging both chemical and electromagnetic interactions between molecules and nanostructured metal surfaces or particles. Depending on factors such as nanoparticle size, shape, and composition, enhancements ranging from 10^4^ to 10^10^ have been reported.^46,47^ We evaluated four different gold particle shapes to identify a suitable SERS enhancer for a 785 nm laser wavelength: nanorods, nanospheres, nanocubes, and nanobipyramids, all known for their potent SERS enhancement properties.^18,48^ Analysis of their respective absorbance spectra (Figure 2A) revealed that among the four types, only nanorods and nanobipyramids demonstrated significant absorbance at 785 nm. Notably, the nanorods exhibited a pronounced absorbance peak near 785 nm, corresponding to their longitudinal plasmon mode, making them highly promising for SERS enhancement at this wavelength. The absorbance spectra of these CTAB-coated gold particles, measured in a solution of deionized water, revealed no significant interference from CTAB itself, as its absorption occurs near 1000 nm. This confirms that the distinct absorbance peaks of the particles are attributed to their unique morphologies and plasmonic properties. Consequently, we selected nanorods for all subsequent SERS measurements.

To understand the binding mode between CaDPA and gold nanorods, we used a DFT model to calculate the binding energy of nonchelated DPA to a linear gold molecule, representing the nanorod surface. Using DPA instead of CaDPA simplifies the modeling process and is relevant as CaDPA is likely to dissociate into DPA in solution. It should also be noted that this diatomic geometry is a simplified model of the gold nanorods used experimentally, as modeling the actual nanorod geometry is infeasible due to the high computational cost involved. We explored several initial geometries to identify the strongest binding mode and discovered that the strongest binding occurs at a geometry when the gold structure is positioned close to the nitrogen atom (Figure 2B, blue), and between the carboxyl groups. This preference for nitrogen binding has been previously demonstrated, for instance, in pyrrolidinone studies by Mdluli et al.^49^ Interestingly, this orientation of DPA and gold is geometrically similar to that of CaDPA, possibly influencing the Raman fingerprint in a similar way as the Ca^2+^ ion in CaDPA. The corresponding bond energy of 81 kJ/mol indicates that the interaction between the molecule and the gold structure is dominated by dipole attraction forces rather than covalent bonds, which typically require larger binding energies (>200 kJ/mol).^50^ Other geometries that exhibited significant, but lower, binding energies include the interaction between the carboxyl and carbonyl groups at 67 kJ/mol, and between the carbonyl group and the pyridine ring structure at 57 kJ/mol (Figure S4). These findings suggest that although the molecular geometry of DPA undergoes some changes upon binding to gold, no additional chemical bonds are formed. Consequently, changes in the Raman spectral fingerprint of DPA due to this binding are likely to be limited. As a final note, although the gold geometry in our calculation is simplified, the binding energies’ scale and relative values should still be comparable with those in the experimental system, even if exact values differ.

### CaDPA from Sonicated Spores Is Distinct from Other Biological Spectral Features

**Figure 3:**
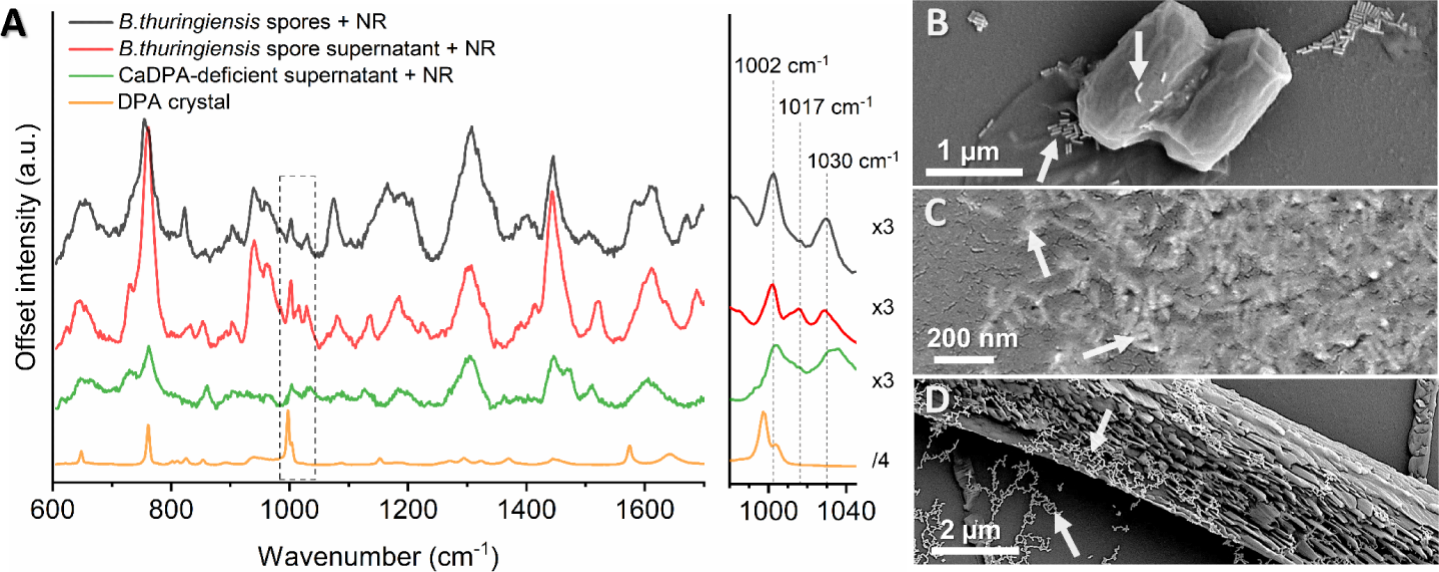
(A) SERS spectra of nanorods (NR) mixed with *B. thuringiensis* spores (black line), supernatant of *B. thuringiensis* (red line), CaDPA-deficient spore supernatant (green line) and DPA crystals (yellow line). The smoothed graphs in the inset of the highlighted region have their values multiplied by a factor of 3 or divided by 4 for easier visualization. Each spectrum represents an average of at least 5 measurements. The three SEM images illustrate the surface morphology of (B) *B. thuringiensis* spores, (C) CaDPA-deficient supernatant, and (D) crystallized nonchelated DPA, all mixed with gold nanorods (highlighted with arrows).

When intact *B. thuringiensis* spores are mixed with gold nanorods, a strong SERS signal emerges, featuring multiple peaks, including one attributed to the ring breathing mode of phenylalanine at 1002 cm^–1^,^51,52^ and a smaller peak at 1030 cm^–1^ (Figure 3A, black line). The observed shift in peak positions compared to previous LTRS measurements (1005 cm^–1^, 1019 cm^–1^ (CaDPA), and 1033 cm^–1^) arises from differences in measurement systems and calibration drifts. Despite the robust signal, the characteristic peak associated with CaDPA (now around 1017 cm^–1^) is notably absent. This absence, however, aligns with theoretical predictions of SERS enhancement, which typically have an effective enhancement distance on the order of a few nanometers.^53^ As illustrated in Figure 3B, the gold nanorods primarily reside on the surface of the spores, thereby enhancing signals predominantly from the outer layers. Even in instances where hotspots form between clustered nanorods, characterized by particularly strong SERS enhancement, the effective distance of SERS is typically less than 20 nm.^54^ This distance is significantly shorter than the approximate 150 nm thickness of the spores’ cortex and coat, and hence, successful detection of a signal from CaDPA requires its release from the spore prior to measurement.

Continuing our investigation, we analyze the SERS spectra of an undiluted sample of dried supernatant, containing the released CaDPA from the disrupted spore stock. In the obtained spectra (Figure 3A, red line), distinct peaks emerge at 1002 cm^–1^, 1017 cm^–1^ (CaDPA) and 1030 cm^–1^. The presence of the 1002 cm^–1^ peak is expected, given the anticipated presence of soluble spore fragments in all supernatant samples. Notably, we are able to clearly detect the characteristic peak of CaDPA from the supernatant, indicating the successful release and identification of the biomarker from disrupted spore using a traditional Raman setup. In contrast, the 1017 cm^–1^ peak is absent in the SERS spectrum of the supernatant from disrupted CaDPA-deficient spores (Figure 3A, green line), even when mixed with gold nanorods (Figure 3C), consistent with previous LTRS measurements (Figure 1E). The peak at 1030 cm^–1^ is rarely mentioned in discussions of spore spectra, yet it appears in reported Raman spectra of whole spores, but not in those of pure CaDPA.^20,55^ Its consistent presence in all previously discussed LTRS spectra, combined with its absence in the spectra of pure DPA (Figure 3A, yellow line), suggests that this peak is unrelated to DPA. Instead, we attribute it to phenylalanine from proteins on the spore surface.^56^

The crystal form of pure DPA further exhibits a prominent Raman peak at 998 cm^–1^, consistent with previously reported spectra,^57^ with a smaller peak appearing at 1005 cm^–1^. Furthermore, it was observed that pure DPA crystallizes into fiber-like structures (Figure 3D), rendering it undetectable outside these crystals, even when mixed with gold nanorods. It is also important to note that the silicon wafer used as the base substrate in this study, nor the CTAB-coated gold nanorods exhibit any significant Raman activity in the 1000–1040 cm^–1^ range, and therefore does not interfere with the discussed peaks (Figure S5). Having established a clear detection of the characteristic CaDPA peak, we next explored the limits of detection and sensitivity of our method.

### CaDPA Signal Remains Visible at Low Concentrations

**Figure 4:**
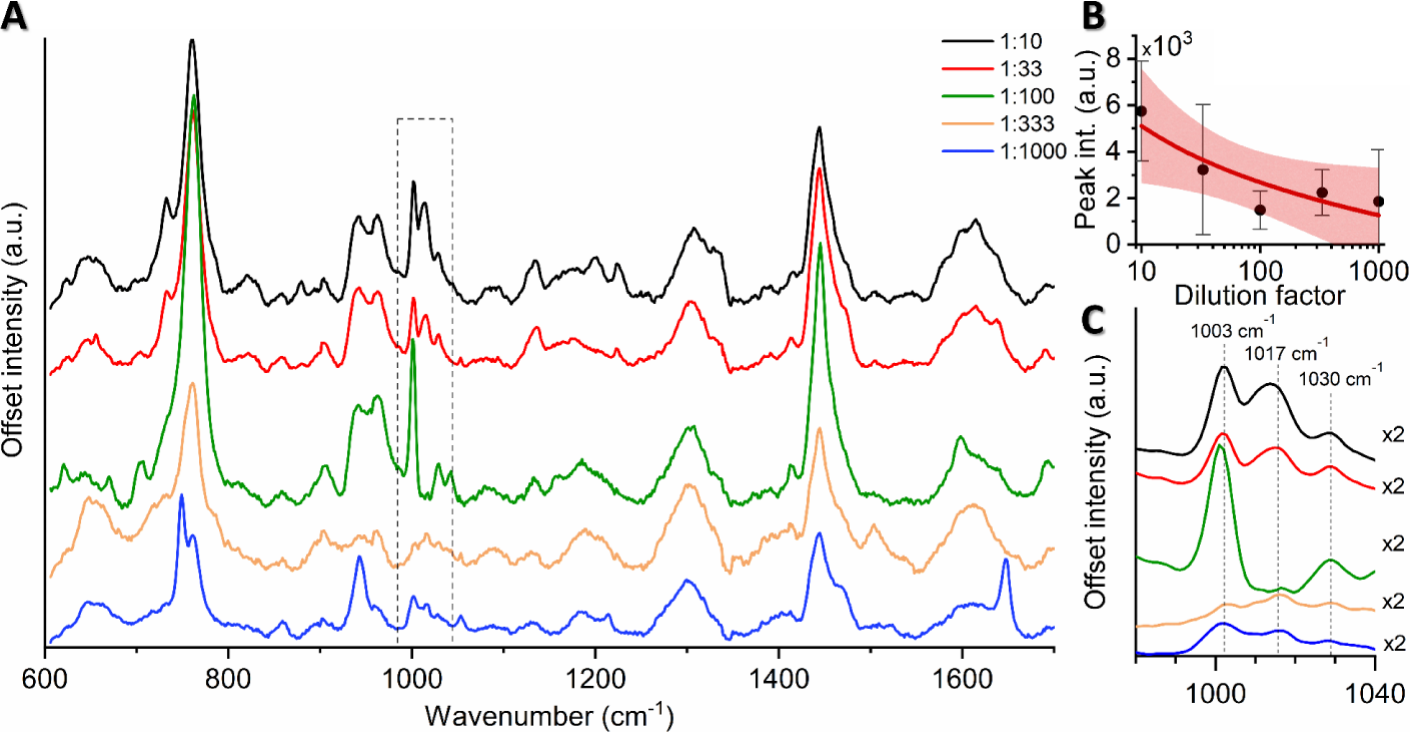
(A) SERS spectra of a dilution series (1:10 to 1:1000) of the spore supernatant, with original spore stock at 10^9^ spores/mL. Each spectrum is an average of at least 15 measurements over 3 droplets, with a minimum of 5 measurements per droplet. (B) Mean intensity of the 1017 cm^–1^ peak, with error bars showing the standard deviation. A best fit curve (red line) with 95% confidence ratio (pale red) shows the general relationship of the peak height and dilution factor. (C) Close up of the region of interest highlighted in (A), with all graphs smoothed and multiplied by a factor of 2 for easier visualization.

To explore the limits of detection, we conducted a dilution series (1:10 to 1:1000) of SERS experiments (using 1 μL droplets) with the CaDPA-containing supernatant, originating from a spore stock of 10^9^ spores/mL (Figure 4A). The 1017 cm^–1^ peak decreased in intensity with lower concentrations (Figure 4B) but remained detectable even at the highest dilution of 1:1000 (Figure 4C). However, we observed significant variations in signal strength between droplets at similar concentrations, likely caused by differences in the drying procedure, which resulted in varying integrations of CaDPA and nanorods. This is manifested by the large standard deviations observed in Figure 4B, with overlapping standard deviations between different dilutions. We also investigated an alternative approach where dried supernatant was layered with nanorods before SERS detection. While this method allowed us to detect a distinctive peak at 1017 cm^–1^ at a concentration corresponding to 10^7^ spores/mL (Figure S6), it presented its own challenges. Some of these include difficulties in achieving efficient overlaps of gold nanorods and biomaterial, as well as increased time consumption due to the need for an extensive search for suitable detection areas. Consequently, we chose to employ the simpler technique of mixing the nanorods with the supernatant, with an accumulation of spore material and nanorods in various “coffee rings” formed during drying. Despite the method’s inherent variability, we detected a distinct peak at concentrations corresponding to 10^6^ spores/mL, demonstrating its robustness.

**Figure 5:**
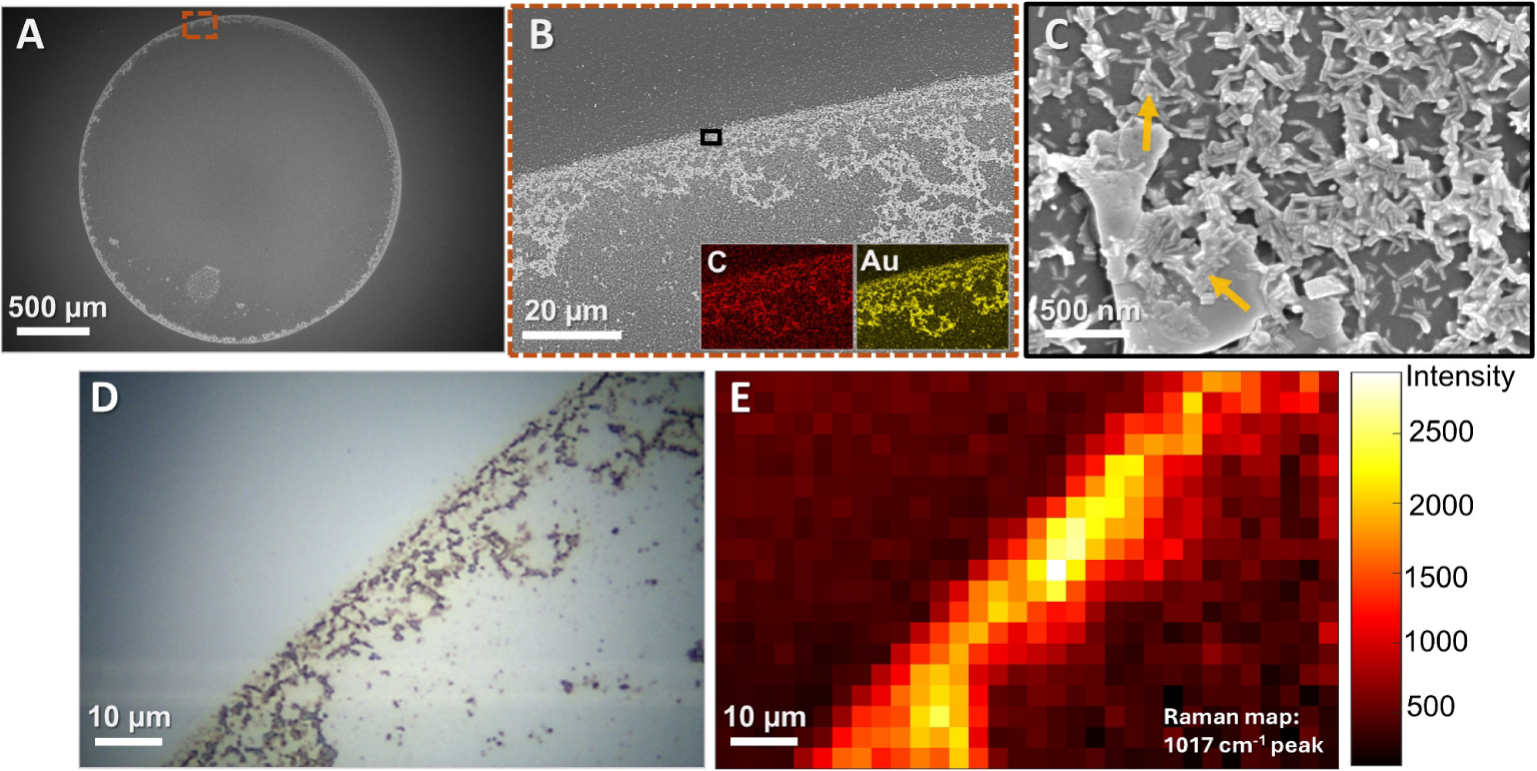
(A)–(C) SEM images of the dried coffee ring of a 1:1000 diluted droplet imaged at increasing magnification, with EDS mapping of gold and carbon as insets in (B). The gold and carbon signals are overlapping significantly, which is further supported in (C) where the agglomeration of Au nanorods (orange arrows) and supernatant is clearly visible. (D) Microscope image of a part of the coffee rings edge. (E) Raman map over the region displayed in (D), showing the intensity of the 1017 cm^–1^ peak.

Under ideal conditions, air-drying a sample droplet results in the formation of a distinct “coffee ring”, which effectively concentrates biomaterials and gold nanorods in close proximity, facilitating their interaction. Figure 5A illustrates this phenomenon with a 1:1000 diluted droplet, where most of the droplet’s contents accumulate along the coffee ring. To further examine the material distribution within this ideal coffee ring, we conducted an EDS mapping of gold (nanorods) and carbon (all organic materials in the supernatant) signals from a specific region marked in the SEM image (Figure 5B). The corresponding maps, shown in the lower insets, clearly demonstrate an overlap between the gold and carbon signals. This overlap is further emphasized in the high-magnification SEM image (Figure 5C), revealing clustered nanorods alongside various forms of spore biomaterials. The significant clustering of nanorods is likely to enhance the SERS signal by creating hotspots within the gaps between them, where particularly strong enhancements can occur.^58,59^

The robustness of our method is further demonstrated by the consistent detection of the 1017 cm^–1^ peak, regardless of the location within the coffee ring from which the SERS signal is collected. To demonstrate this uniformity, we Raman mapped the area depicted in Figure 5D for the 1017 cm^–1^ peak. The results (Figure 5E), show the presence of the CaDPA peak throughout the coffee ring, with no significant signals detected at other locations. However, the appearance of this “coffee ring” is a complex procedure and is influenced by multiple factors. These include the size and relative concentration of organic material and nanoparticles in the solute, surface hydrophobicity, surface impurities or defects and environmental factors affecting the droplet’s evaporation, such as temperature, pressure and humidity.^60^ The effect of concentration, different cleaning methods and pressure is shown in Figures S7 and S8, where different conditions lead to the formation of various deposition patterns, including some with multiple rings or material being spread out. Despite these large variations in deposition patterns, we consistently detect CaDPA, as is further demonstrated by the replicate measurement series in Figure S9.

### Detecting CaDPA from 10^3^ Spores/mL

We next aimed to determine the detection limit of our method in realistic spore suspensions at low concentrations, rather than dilution from high-concentration experiments, which is common in literature. We prepared and sonicated spore suspensions at concentrations of 10^4^, 10^3^ and 10^2^ spores/mL, confirming the spore concentrations by drop counting prior to each experiment. Consistent with earlier findings, where ∼75% of spores released CaDPA, probe sonication in these experiments damaged over 80% of the spores, preventing their germination on agar plates postsonication. We also employed smaller suspension droplets of 0.1 μL for all SERS measurements in this series.

**Figure 6:**
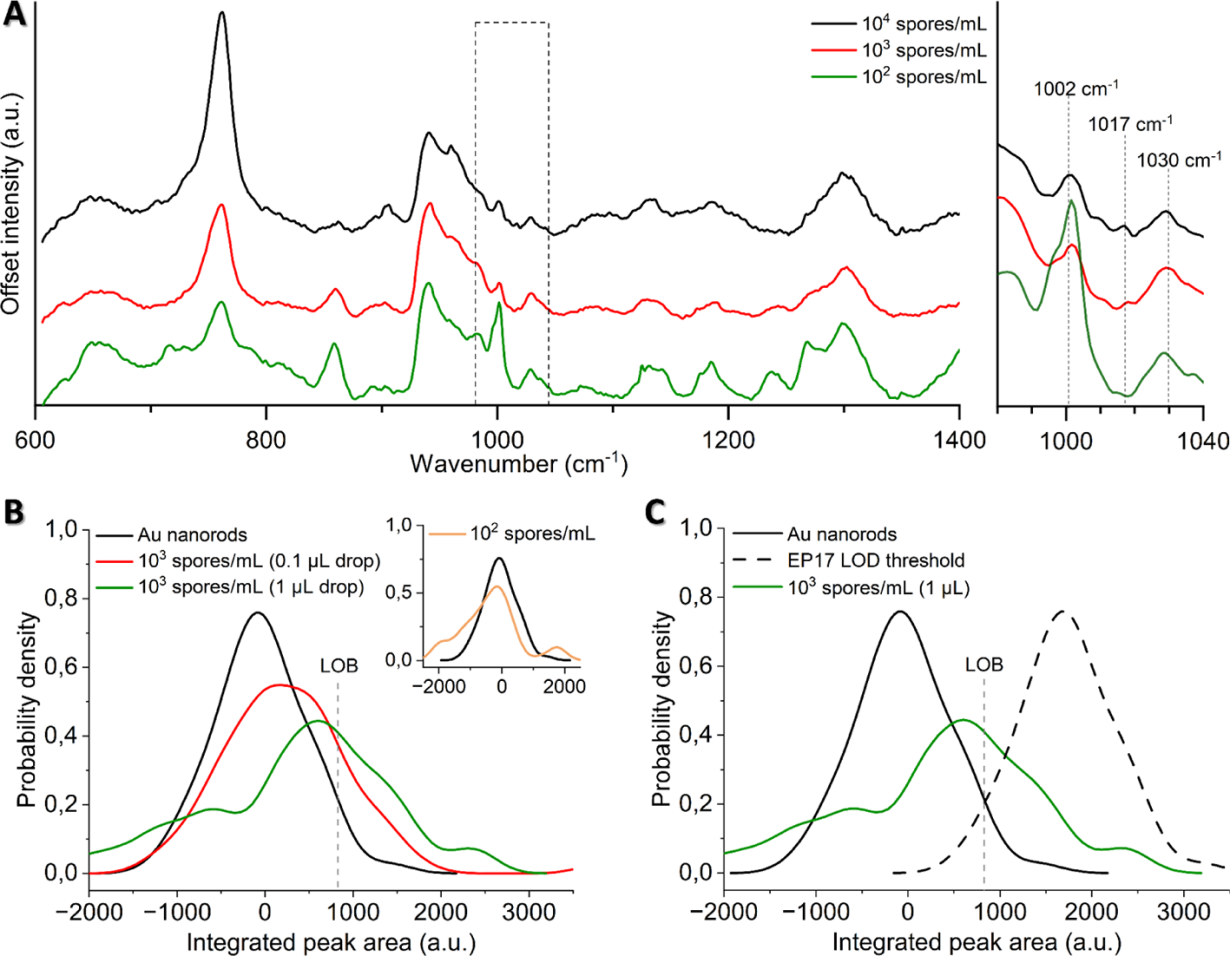
(A) Smoothed SERS spectra of sonicated spore suspensions at concentrations of 10^4^, 10^3^ and 10^2^ spores/mL. The 1017 cm^–1^ peak remains detectable down to 10^3^ spores/mL. All spectra are an average of at least 10 individual spectra from 4 different droplets. (B) Kernel density plots of the peak area between 1014 and 1020 cm^–1^ from at least 60 spectra, for a control of Au nanorods and 10^3^ spores/mL at different droplet volumes. The inset highlights 10^2^ spores/mL (0.1 μL droplet) against the nanorod control. (C) Kernel density plots showing the EP17 threshold for LOD, with the limit of blank indicated.

While the resulting coffee rings were less distinct (Figure S10), the characteristic 1017 cm^–1^ peak (Figure 6A) remained detectable down to 10^3^ spores/mL, but not at 10^2^ spores/mL. To further assess and quantify peak detection at these low concentrations, we evaluated over 60 spectra across five 0.1 μL droplets and constructed kernel density plots of peak areas between 1014 cm^–1^ and 1020 cm^–1^ for 10^3^ spores/mL, 10^2^ spores/mL and a nanorod control (Figure 6B). The control and 10^2^ spores/mL distributions both centered around zero, consistent with noise levels. In contrast, the 10^3^ spores/mL distribution showed a clear shift, with 19% of all data above the LoB, indicating a measurable signal. Increasing the droplet size to 1 μL further amplified this signal, yielding a total of 33% above the LOB, demonstrating how droplet volume optimization can enhance detection sensitivity. While these results are promising and show the potential of this method for low-concentration spore detection, they also reveal variability in peak area measurements, indicating the need for further optimization to improve reproducibility. However, the substantial increase in peak area for 1 μL droplets suggests that, with further refinement, a sensor based on this methodology could achieve the sensitivity and consistency required to meet industrial LOD standards at low concentrations. For instance, a 3-fold improvement in signal detection at 10^3^ spores/mL (Figure 6C) could enable the method to meet the EP17 threshold for LOD—a criterion set by the Clinical and Laboratory Standards for establishing analytical sensitivity and detection limits in diagnostic methods.^32^ While the inherent variability of SERS may limit its ability to fully achieve industrial-level LOD, its potential as a rapid and efficient preliminary screening tool remains substantial, offering valuable insights and facilitating timely follow-up confirmatory tests.

Furthermore, assuming a homogeneous distribution of CaDPA in the supernatant, each 0.1 μL droplet at 10^3^ spores/mL contains CaDPA equivalent to only 10% of a single spore. Combined with the fact that each SERS measurement captures data from only a small portion of the coffee ring (Figure S11), this indicates a sensitivity well below the level of a single spore. As such, we conclude that this method currently achieves subspore sensitivity, with the capability of detecting concentrations as low as 10^3^ spores/mL. Since most studies do not adhere to the EP17 standard, this detection represents an order of magnitude improvement compared to previously reported LODs for CaDPA detection, with a sensitivity 3 orders of magnitude higher than earlier methods (see Table S1 for details). Furthermore, this detection limit is below typical infectious doses of pathogenic spores, like *B. cereus* in food poisoning and *B. anthracis* in anthrax, which can cause infections at levels as low as 10^4^ CFU,^24,28^ demonstrating its relevance for pathogen detection. As an example of the method’s broader applicability, its performance was next evaluated in the more complex biological matrix of milk.

### Detection of Spores in Milk

Milk and dairy products are routinely monitored to prevent contamination in production lines, with spore contaminations from *Bacillus* species being a major hazard. However, detecting spores in milk presents a practical challenge due to milk’s complex mixture of proteins, lipids, and sugars. To evaluate our method’s effectiveness in this context, we spiked commercial milk samples with spores at an initial concentration of 10^6^ spores/mL. To reduce interference from milk components, we dilute the sample 1:1000 and use a similar detection method as previously, with 0.1 μL droplets.

**Figure 7:**
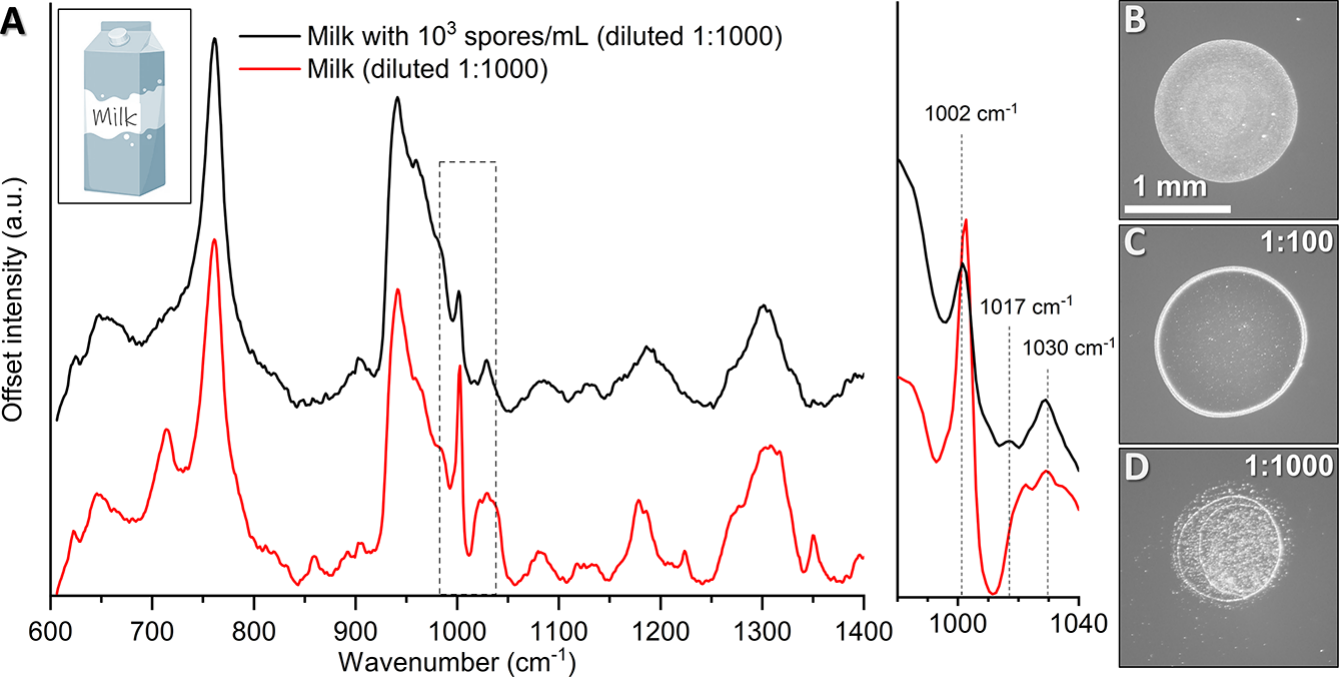
(A) Smoothened SERS spectra comparing milk containing 10^3^ spores/mL (diluted 1:1000) with spore-free milk, demonstrating a detectable CaDPA peak at 1017 cm^–1^. The spectra represent the average of 15 measurements across 3 different droplets. (B), (C) and (D) show photographs of dried 0.1 μL droplets used in the experiment, with (B) representing the undiluted sample at 10^6^ spores/mL, (C) a 1:100 dilution in distilled water, and (D) a 1:1000 dilution.

After dilution, we successfully demonstrate the detection of the CaDPA peak at 1017 cm^–1^ of a sample corresponding to 10^3^ spores/mL (Figure 7A, black), with sensitivity and peak area matching results from the water-based experiments in Figures 6A,B and S12. When comparing the spectra to a control of milk (Figure 7A, red), some differences are apparent, including a broad peak between 1020–1040 cm^–1^ only present in the control. The differences in spectral composition likely arise from the complex matrix of milk, where the proximity of different molecules to the gold nanorods leads to variations in the recorded spectra.

Using this simple and nonoptimized protocol we show a practical detection limit of 10^6^ spores/mL, in range of typically reported contamination levels for *B. cereus* spores in food (10^5^ to 10^8^ CFU/g).^28^ Currently, this detection method is limited by the necessity of sample dilution (Figure 7B–D), to reduce interfering milk components and achieve a clear signal. Refining the methodology, for instance, by purifying spores from milk components through enzymatic digestion and centrifugation, may alleviate this issue. Utilizing stronger plasmonic enhancers or optimizing droplet volumes or drying conditions may also further enhance both the detection limit and its reproducibility, thus increasing the method’s applicability in industrial settings.

## Conclusions

In this study, we developed a sensitive, simple and specific label-free method for detecting bacterial spores using SERS. The method leverages coffee rings formed during drop drying, utilizing a mixture of gold nanorods and the supernatant from disrupted spores for enhanced detection. Using probe sonication, we show that ∼75% of spores release their CaDPA. By targeting the distinctive biochemical signature of CaDPA at 1017 cm^–1^, we achieved subspore level sensitivity with an experimental detection limit of 10^3^ spores/mL. The specificity of our method was validated by using a CaDPA-deficient mutant strain, ensuring that the detected 1017 cm^–1^ signal originated from CaDPA rather than other organic material. Importantly, the demonstrated detection of 10^3^ spores/mL is below typical infectious doses of pathogenic spores, like *B. cereus* in food poisoning and *B. anthracis* in anthrax, which can be infectious at levels as low as 10^4^ CFU.^24,28^ Finally, we demonstrated the applicability of our method by detecting the CaDPA peak in a real-world sample of milk spiked with 10^6^ spores.

Our study also emphasizes the importance of controlling the sample preparation steps in achieving enhanced detection sensitivity. Under optimized conditions, the formation of the coffee ring demonstrates a promising approach for developing sensors that meet EP17 LOD standards for low and relevant spore concentrations, with applications in fields such as environmental monitoring and food industry

## Supporting Information Available

The Supporting Information includes detailed experimental data and analyses. This includes a TEM image of gold nanorods with size estimates; a HPLC chromatogram for DPA detection; Raman spectra of spores and controls; additional SERS spectra of spore supernatants in dilution series; a DFT simulation of DPA binding to a gold dimer; a kernel density plot for signal detection in milk; images of dried droplets under varying conditions; and a schematic of the method for sensitivity determination. Comparative analysis of detection limits in the literature is also provided. (PDF)
