# Supplementary material for: Ultra-Sensitive Detection of Bacterial Spores via SERS
Source: ACS Sens. 2025 Jan 23;10(2):1237–48. doi: 10.1021/acssensors.4c03151 (PMC11877637; doi:10.1021/acssensors.4c03151)
Supplement: Supplementary file 1 — se4c03151_si_001.pdf [file se4c03151_si_001.pdf]

Supporting information

Ultra-Sensitive Detection of Bacterial Spores via SERS

Jonas Segervald[1,&], *Dmitry Malyshev*[1,&], *Rasmus Öberg*[1], *Erik Zäll*[1], *Xueen Jia*[1], *Thomas Wågberg*[1,2]*, *and Magnus Andersson*[1]*

[1]Department of Physics, Umeå University, Umeå SE-901 87, Sweden

[2]Wallenberg Initiative Materials Science for Sustainability, Department of Physics, Umeå University, Umeå SE-901 87, Sweden

[&] Contributed equally to this work.

* Corresponding authors: thomas.wagberg@umu.se and magnus.andersson@umu.se

Table of Contents

Figure S1: TEM image of Au nanorods with estimated length and diameter.
Figure S2: HPLC chromatogram comparing pure DPA at 10 µM and 100 µM with a diluted supernatant from the original spore stock (10^9 spores/mL).
Figure S3: LTRS measurements of 30 spores, verifying the release of CaDPA.
Figure S4: DFT simulation of DPA binding to a gold dimer, with optimized geometries between A) the carbonyl and carboxyl groups and B) the carbonyl group and pyridine ring structure.
Figure S5: Raman spectra of intact spores, CTAB-coated gold nanorods and a silicon wafer.
Figure S6: SERS spectra of spore supernatant in a dilution series from 1:3 to 1:100 of the 10^9 spores/mL stock, with Au nanorods applied to dried droplets.
Figure S7: Photographs of dried droplets from a dilution series (1:10 to 1:1000) of the supernatant mixed with Au nanorods.
Figure S8: Images of dried droplets from the 1:333 dilution of the 10^9 spores/mL stock mixed with Au nanorods, under different surface cleaning and pressure protocols.
Figure S9: SERS spectra of a replicate dilution series (1:10 – 1:1000) of the spore supernatant from the original stock (10^9 spores/mL).
Figure S10: Photographs of dried 0.1 µL droplets from 10^4, 10^3 and 10^2 spores/mL.
Figure S11: A schematic illustrating how the method's sensitivity was determined.
Table S1: Comparative analysis of the limit of detection in relevant literature, detailing extraction methods and DPA sensitivity. References correspond to citations in the main manuscript.
Figure S12: Kernel density plots of 60 measurements from 5 droplets of gold nanorods, 10^3 spores/mL and a 1:1000 dilution of milk spiked with 10^6 spores/mL.

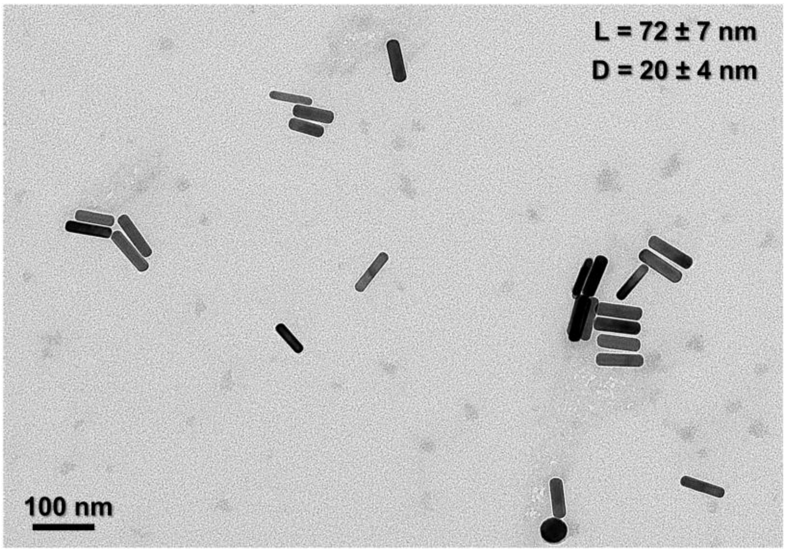

Figure S1. TEM image of the gold nanorods (NR-20-780-50) used in the SERS experiments. The mean length of n=25 measurements and diameter (n=20) out of nanorods are presented in the image, with respective standard deviation.

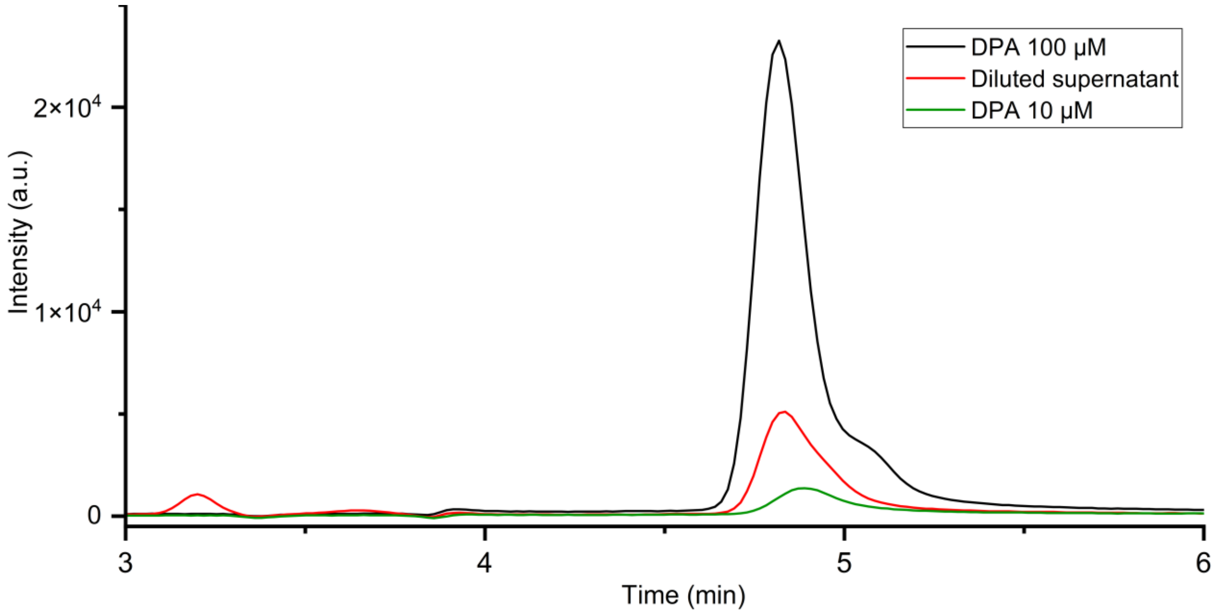

Figure S2. HPLC chromatogram comparing pure DPA at 10 µM and 100 µM with a diluted supernatant. The peak confirms the presence of CaDPA in the supernatant, and its area corresponds to a concentration of 25 µM in the diluted sample, which equates to 2.5 mM in the sonicated spore stock.

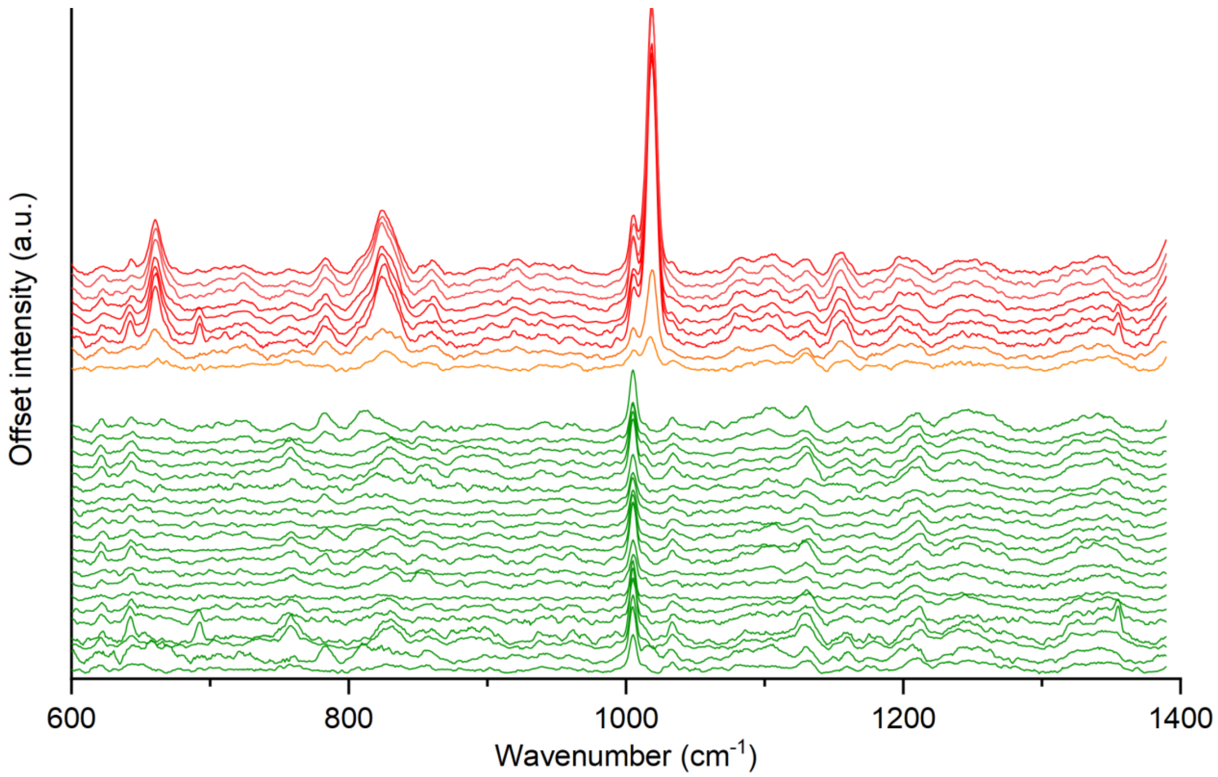

Figure S3. Individual Raman spectra of 30 spores after sonication. Seven spores (red) retain their internal CaDPA. Two spores partially release CaDPA (orange) and 21 spores fully release CaDPA. This corresponds to a CaDPA release of approximately 75 %.

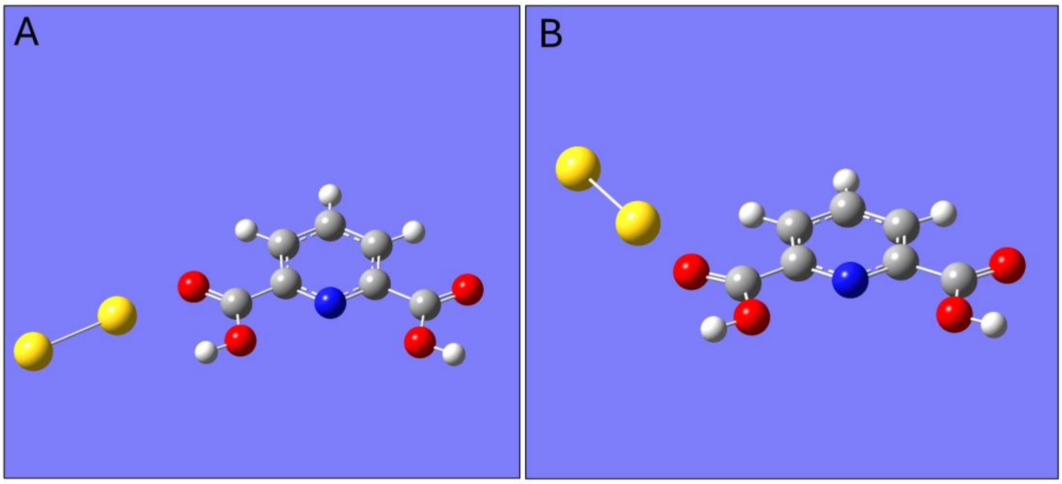

Figure S4. DFT simulation showing optimized geometries of DPA binding to gold dimer A) between the carbonyl and carboxyl group, B) as well as between the carbonyl group and pyridine ring structure. The white, grey, blue, red, and yellow spheres correspond to hydrogen, carbon, nitrogen, oxygen, and gold respectively.

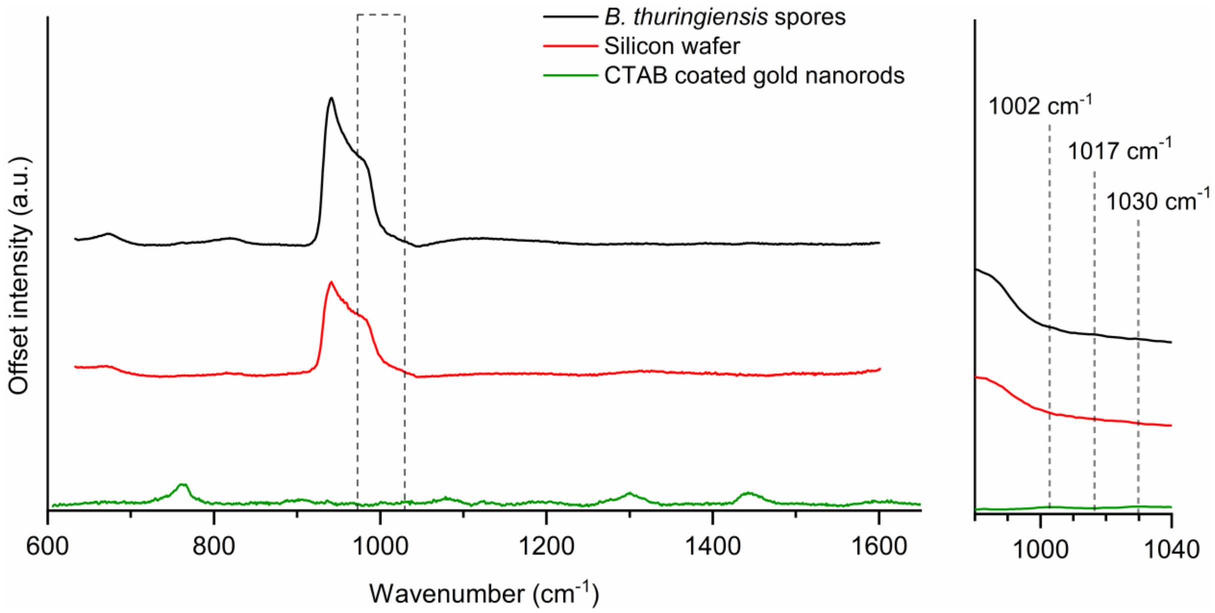

Figure S5. Raman spectra of intact spores, CTAB-coated gold nanorods and a silicon wafer. No unique Raman peaks are observed when measuring on spores or silicon wafer directly, with only the broad band at 960 cm^{-1} from the silicon wafer. Each spectrum represents an average of at least 4 replicates.

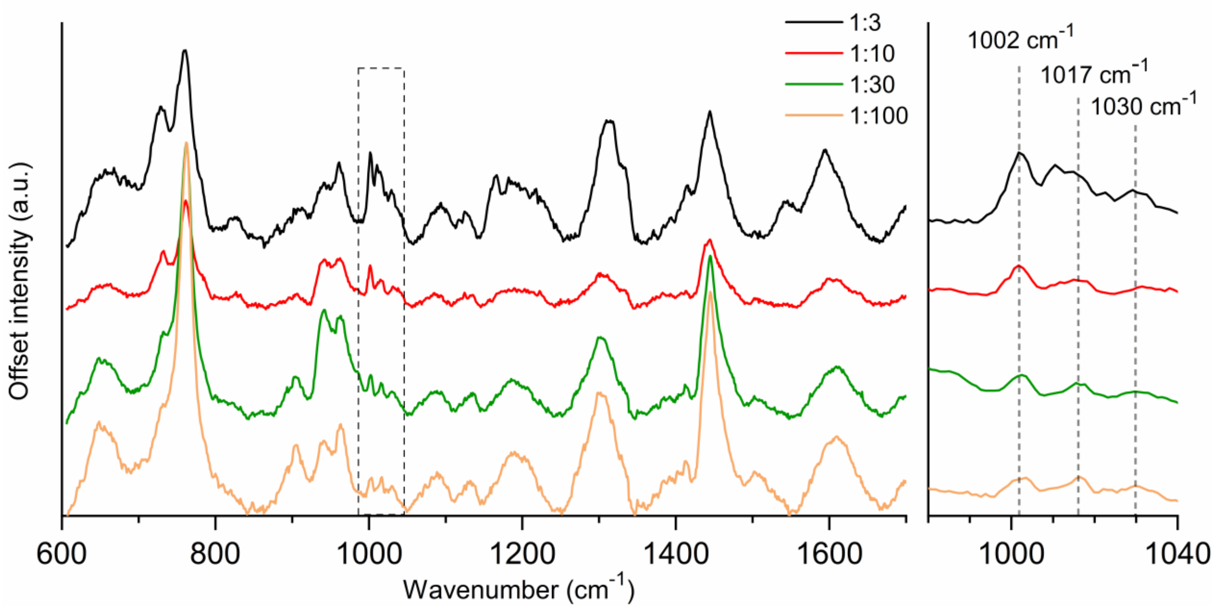

Figure S6. SERS spectra of a dilution series (1:3 – 1:100) using 1 µL droplets from a supernatant suspension corresponding to 10^{7} spores/mL, covered by nanorods. The 1017 cm^{-1} peak remains visible with a 100-fold dilution of the supernatant. Each spectrum is an average of at least 5 replicates.

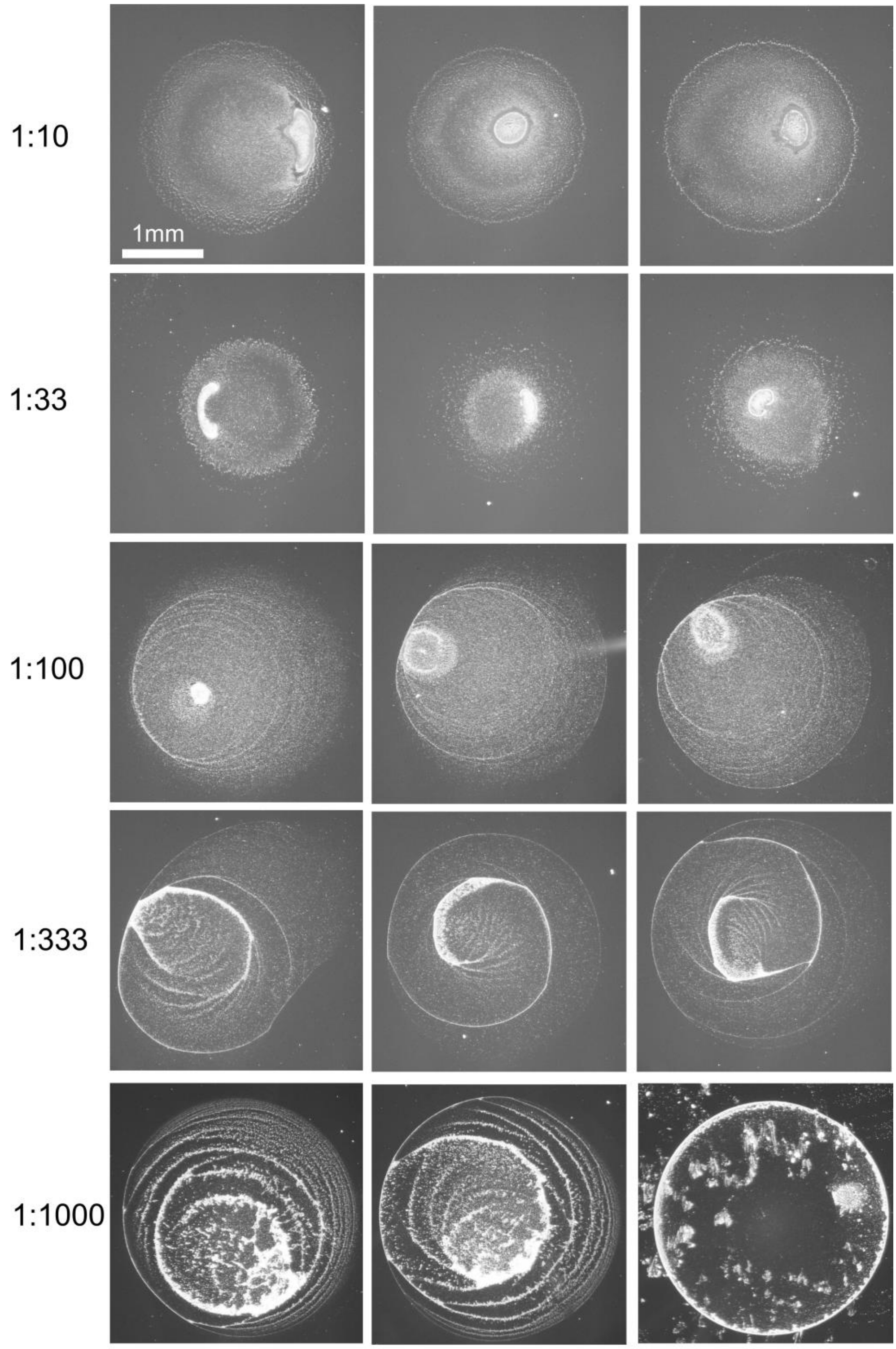

Figure S7. Photos of the drops from the dilution series of the supernatant ranging from 1:10 to 1:1000 mixed with gold nanorods, starting from an original solution of 10^9 spores/mL. The deposition patterns of the droplets in the measured dataset vary depending on the dilution of the extracted CaDPA. Note that the final 1:1000 image was contaminated during storage and therefore slightly differs from Figure 5A.

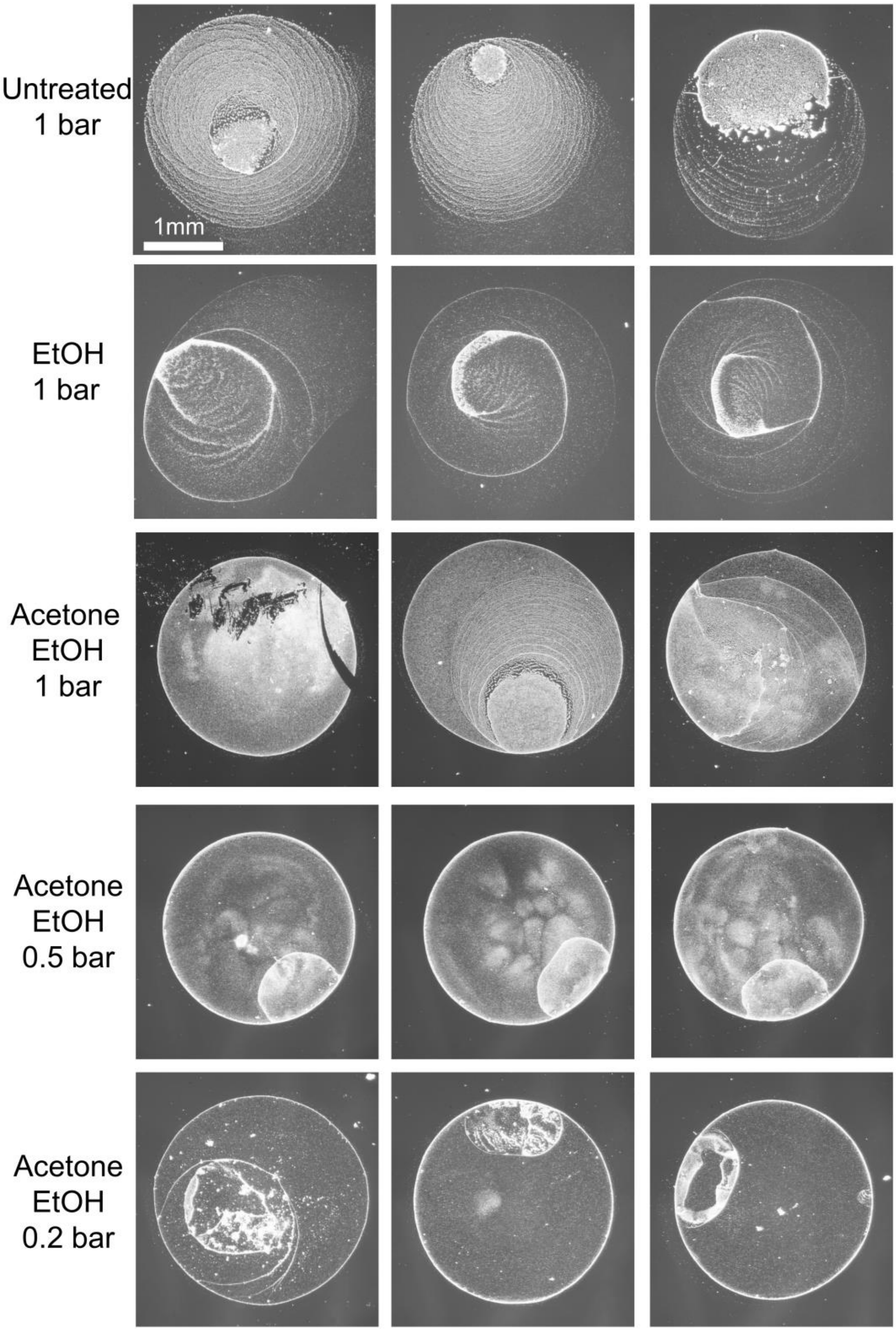

Figure S8. Photos of dried droplets of the 1:333 dilution (equivalent to 3×10^6 spores/mL) mixed with gold nanorods. The dried droplets have different shapes and material distributions depending on the surface treatment (no treatment, ethanol wash, or ethanol and acetone wash), as well as air pressure (affecting the drying speed). All samples were rinsed in distilled water as a final step.

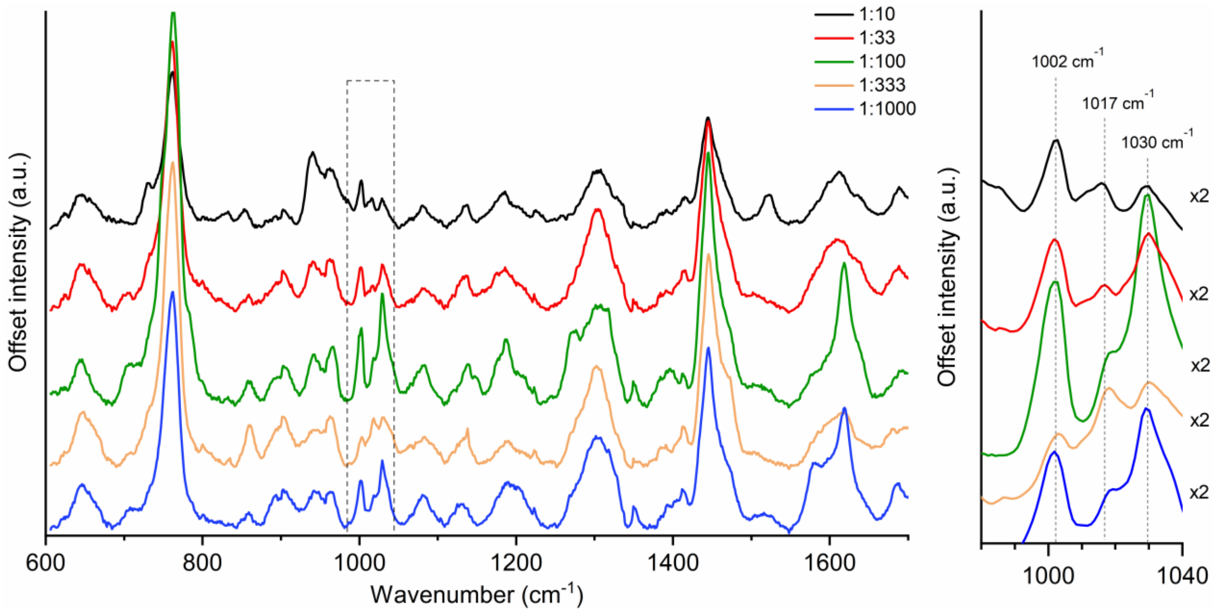

Figure S9. SERS spectra of a second dilution series (1:10 – 1:1000) of the spore supernatant, with original spore stock at 10^9 spores/mL. Each spectrum is an average of at least 15 measurements over 3 droplets, with a minimum of 5 measurements per droplet.

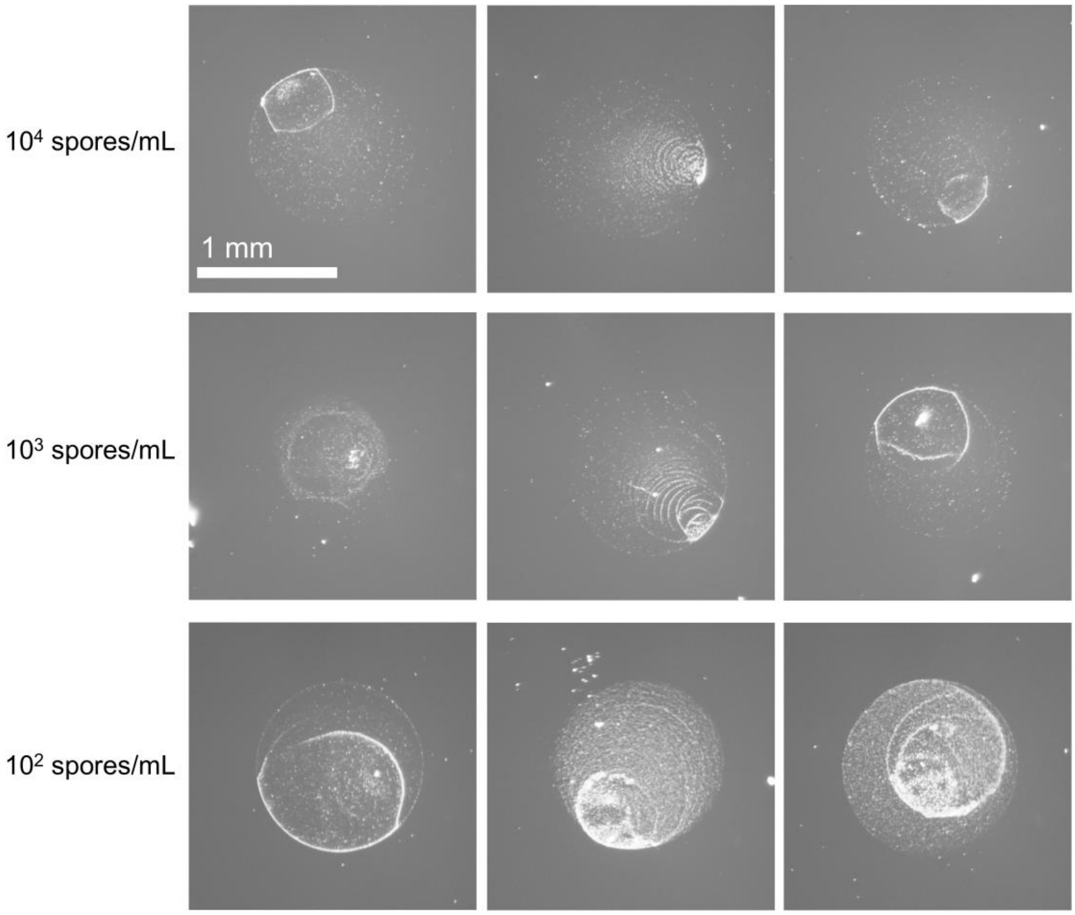

Figure S10. Photographs of dried 0.1 µL droplets of the supernatant from sonicated concentrations of 10^4, 10^3 and 10^2 spores/mL mixed with gold nanorods.

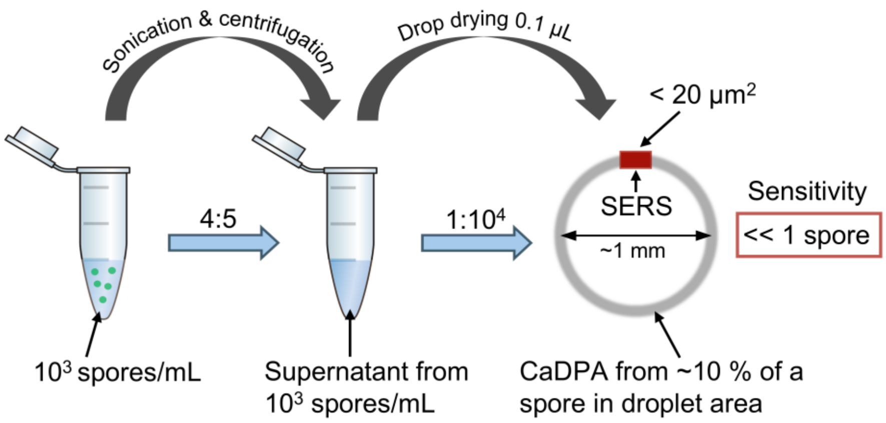

Figure S11. A schematic illustration of how the method's sensitivity was determined. Each droplet contains CaDPA corresponding to ~10% of a single spore. As only a fraction of the droplet's area (< 20 µm^2) is scanned during each SERS measurement, the resulting sensitivity is demonstrated at levels significantly below that of a single spore.

Table S1: Comparative analysis of relevant literature, with focus on their estimated and actual method's limit of detection, their method of CaDPA extraction and sensitivity of pure DPA or CaDPA. The substances presented in the row for CaDPA extraction are dodecylamine (DDA) and nitric acid (HNO_3).

**Author (year)**	Farquharson et al (2004)	Zhang et al (2005)	Cowcher et al (2013)	Jiang et al (2023)	Our work (2024)
**Extraction method of CaDPA**	Mixed with 100 μl DDA (50mM)	Sonication in HNO_3 (20mM)	Sonication in HNO_3 (40mM)	Sonication in HNO_3 (40mM)	Probe sonication
**Experimental LOD (CaDPA)**	10^6 spores (based on a mass of spores)	~10^7 spores/mL (estimated based on dilution)	~2×10^6 spores (peak area comparison with pure DPA series)	1.2×10^4 spores/mL (colony counting of diluted sample)	10^3 spores/mL (colony counting pre-sonication of sample)
**Calculated LOD (CaDPA)**	~10^4 spores (estimated based on S/N ratio)	N/A	N/A	N/A	N/A
**Sensitivity (Pure DPA)**	N/A	N/A	~ 1000 spores (in a 200 μL sample)	~ 100 spores (in a 20 μL sample)	N/A
**Sensitivity (CaDPA)**	N/A	2.6×10^3 spores (in a 0.2 μL sample)	N/A	~ 100 spores (in a 20 μL sample)	<< 1 spore (in a 0.1 μL sample)
**Reference**	[22]	[17]	[23]	[24]	This study

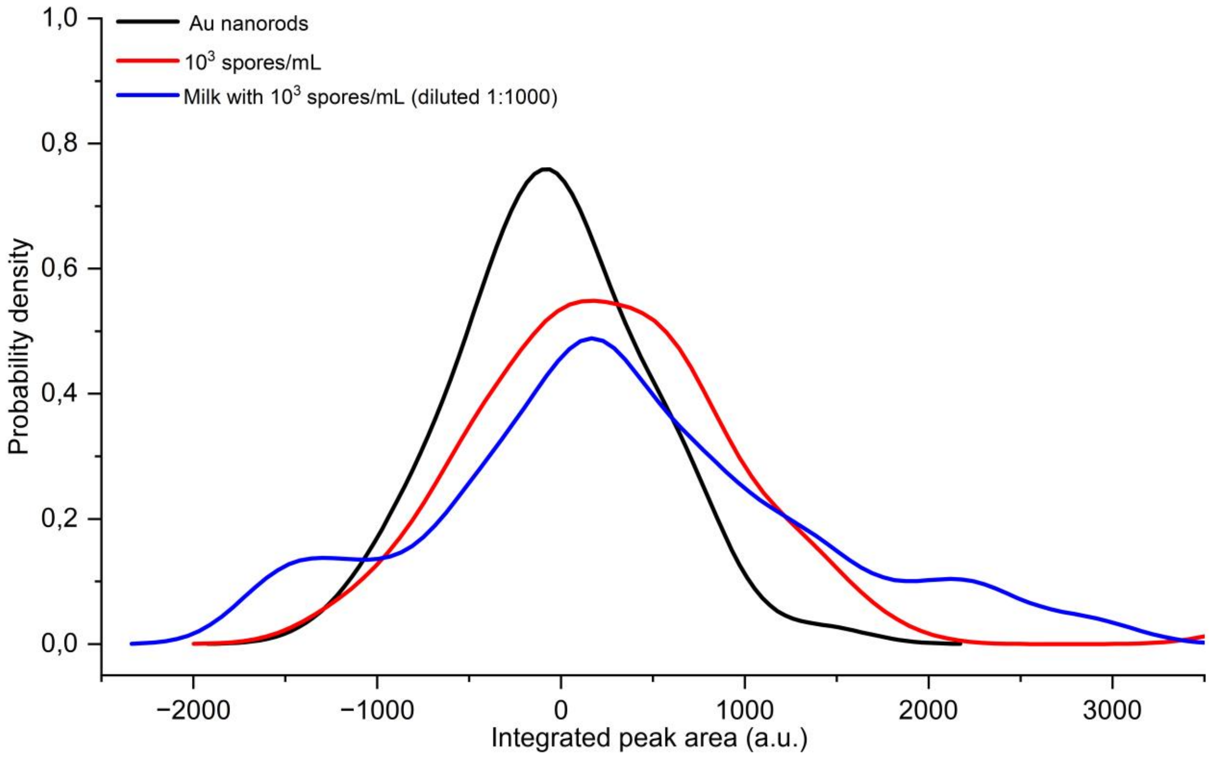

Figure S12. Kernel density plots of a minimum 60 measurements across 5 different 0.1 µL droplets, showing the control of gold nanorods, and the spore concentration of 10^3 spores/mL in water versus a similar concentration in milk (diluted 1:1000).
